# Reliability-Aware Cooperative Node Sleeping and Clustering in Duty-Cycled Sensors Networks

**DOI:** 10.3390/s18010127

**Published:** 2018-01-04

**Authors:** Jeungeun Song, Yiming Miao, Enmin Song, M. Shamim Hossain, Mohammed F. Alhamid

**Affiliations:** 1 School of Computer Science and Technology, Huazhong University of Science and Technology, Wuhan 430074, China; jsong@hust.edu.cn (J.S.); yimingmiao@hust.edu.cn (Y.M.); 2 Department of Software Engineering, College of Computer and Information Sciences, King Saud University, Riyadh 11543, Saudi Arabia; mohalhamid@ksu.edu.sa

**Keywords:** beaconless geographic routing, cooperative communication, duty cycle, energy efficiency, reliability-aware

## Abstract

Duty-cycled sensor networks provide a new perspective for improvement of energy efficiency and reliability assurance of multi-hop cooperative sensor networks. In this paper, we consider the energy-efficient cooperative node sleeping and clustering problems in cooperative sensor networks where clusters of relay nodes jointly transmit sensory data to the next hop. Our key idea for guaranteeing reliability is to exploit the on-demand number of cooperative nodes, facilitating the prediction of personalized end-to-end (ETE) reliability. Namely, a novel reliability-aware cooperative routing (RCR) scheme is proposed to select k-cooperative nodes at every hop (RCR-selection). After selecting k cooperative nodes at every hop, all of the non-cooperative nodes will go into sleep status. In order to solve the cooperative node clustering problem, we propose the RCR-based optimal relay assignment and cooperative data delivery (RCR-delivery) scheme to provide a low-communication-overhead data transmission and an optimal duty cycle for a given number of cooperative nodes when the network is dynamic, which enables part of cooperative nodes to switch into idle status for further energy saving. Through the extensive OPNET-based simulations, we show that the proposed scheme significantly outperforms the existing geographic routing schemes and beaconless geographic routings in wireless sensor networks with a highly dynamic wireless channel and controls energy consumption, while ETE reliability is effectively guaranteed.

## 1. Introduction

Due to the limitation of sensor nodes in wireless sensor networks (WSNs), we have to pay more attention to the reduction of node energy consumption and prolonging of network life. Meanwhile, due to the high dynamics of wireless links, reliable data transmission is also indispensable. The duty-cycled sensor networks provide a new perspective for improving the performances of multi-hop cooperative sensor networks, especially energy efficiency and reliability assurance, which rely on optimal relay assignment, relaying candidate selection and cooperative communication [1,2]. In this paper, we first propose a novel routing scheme with high energy efficiency and high fault tolerance, named the reliability-aware cooperative routing (RCR), which is based on RCR-selection and RCR-delivery. We consider energy-efficient cooperative node sleeping and clustering problems in cooperative sensor networks where clusters of relay nodes jointly transmit sensory data to the next hop [3]. In addition, the key idea for guaranteeing reliability is to exploit the on-demand number of cooperative nodes (RCR-selection), facilitating the prediction of personalized end-to-end (ETE) reliability. Thus, a  probabilistic ETE reliability model is built to compute an optimal duty cycle for RCR in the online manner. Besides the sensory data transmission strategy (RCR-delivery) between cooperative nodes with the minimal number under the condition of the given requirement of ETE reliability, it also controls the energy consumption.

In RCR-selection, a source node initializes the network by a broadcast probe (PROB) message. Then, it transmits the probe message to find a first-hop reference node (RN). The RN selects *k*-cooperative nodes according to the residual energy and distance from its neighbors and calculates the location of the next-hop RN. The sensory data are transmitted by the cooperative node with the highest residual energy in every hop using a probe message, until the sink node receives the data packet. After the network has run for a certain period, the source node will resend a probe message to find a new set of cooperative nodes since the energy of nodes is reduced.

In RCR-delivery, RNs will allocate the same length back-off delay to each of their cooperative nodes. A cooperative node with more residual energy will be assigned a time slot with higher priority. After predetermining the time slots, the sensory data will be broadcast by a packet holder during the data delivery phase. When receiving a data packet, each cooperative node fires its timer through the predetermined back-off delay. The one whose timer expires first, which is usually a cooperative node with the highest priority, will continuously broadcast the data packet. At the same time, the other cooperative nodes will notice that another node has forwarded the data packet by snooping the data message and quit the contention process immediately.

Compared to the relaying candidate selection [4] and potential forwarders (PFs) selection of conventional beaconless geographic routing [5], the RCR-selection has the following advantages:

- The PFs’ selection strategy of traditional beaconless geo-routing relies on the shape and size of a certain sensory data forwarding region. However, the number of PFs in the forwarding region for each relay node (RN) is uncertain, and it fluctuates from hop to hop. Although some nodes have a relatively large number of PFs, the ETE reliability still cannot be guaranteed because some “bottleneck” hop has very few PFs. Compared to the beaconless geo-routing, the RCR-selection always ensure *k* cooperative nodes at each hop, to improve personalized ETE reliability.

Further, compared to the data transmission and forwarding strategy of the conventional beaconless geographic routing, the RCR-delivery performs better, as shown below:

- Similar to the time division multiple access (TDMA-like) approach, the time slots of RCR-delivery are allocated by reference nodes (RN) in a centralized scheme before data transmission from one hop to the next hop. Simulation results showed that every time slot should be as short as possible. However, we should guarantee that PFs can receive data packets from other higher priority PFs before their timer expires. Thus, the value of each predetermined time slot should be long enough to accommodate the hop delay at least. Therein, a hop delay denotes the elapsed time from the moment a sensor node sends a data packet (to its farthest neighbor node) to the moment when a neighbor node receives it. Different from the three hand-shaking mechanism of beaconless geo-routing, the RCR-delivery performs with lower delay under the same packet delivery rate in order to avoid collision.
- In traditional beaconless geo-routing protocols, the current packet holder (PH) typically broadcasts a probe message to PFs and waits for a reply. After receiving the first reply, which shows that a PF will be the next packet holder, the current PH transmits the data packet to the candidate by unicast and release memory. Different from such a high cost data forwarding scheme, the RCR-delivery does not determine which packet holder will be the next candidate. In addition, due to the centralized forwarding delays allocation of cooperative nodes, the collision ratio of RCR-delivery can be ignored.
- In order to achieve a load balancing among in RCR, RN assigns priority to every cooperative node according to its residual energy information. In addition, almost all of the existing traditional beaconless geo-routing schemes cannot effectively guarantee network lifetime [6].
- Due to the dynamical scheduling of duty cycles of cooperative nodes, the RCR-delivery can guarantee on-demand ETE reliability while achieving energy efficiency. By comparison, almost none of the existing traditional beaconless geo-routing schemes can guarantee energy efficiency when transmitting sensory data because all of the PFs must be awake and stay active all the time to attend the election [7].

Generally, wireless sensor networks are powered by energy-limited batteries, which are almost impossible to charge or replace [8]. A common way to extend the lifetime of WSNs is to select one or a few sensors to remain in active status [9] while allowing others to sleep [10]. In RCR-delivery, we try to transmit the sensory data between cooperative nodes with a minimal number of hops for a given requirement for ETE reliability and energy saving. Thus, in this paper, we build an ETE reliability model with reliability-aware cooperative nodes to investigate the following problems: the relationship between the on-demand ETE reliability [11] and the number of cooperative nodes for a certain average node density, link failure ratio and duty cycle [12] at each hop; and the relationship between the on-demand ETE reliability and the duty cycle of cooperative nodes for certain *k* numbers of cooperative nodes at each hop. Furthermore, under the condition of a certain duty cycle, we design a sleep scheduling algorithm to achieve load balance and energy saving of cooperative nodes and prolong network lifetime. In our scheme, the cooperative nodes with lower residual energy have more chance to enter sleeping status. Table 1 gives the expression of the main notations.

This paper is arranged as follows. The related works are presented in Section 2. The network model for analysis of the RCR scheme is given in Section 3. The on-demand ETE reliability model, which implies the key design of RCR, is introduced in Section 4. In Section 5, the RCR algorithm is presented in detail. The performance evaluation of RCR is provided in Section 6. Finally, the work is concluded in Section 7.

## 2. Related Works

Our work is closely related to the geographic routing scheme, beaconless geographic routing, cooperative communications and ETE reliability QoS provisioning in WSNs. In the following, we give a brief review of related works in these aspects, as shown in Table 2.

### 2.1. Geographic Routing

Due to good scalability and efficiency, geographic routing is an attractive localized routing for WSNs. Most existing geo-routing protocols require every fixed node in the network to broadcast its accurate position information, so that all its direct neighbors can precisely locate the node. The greedy perimeter stateless routing (GPSR) [14] is a representative stateful geo-routing protocol wherein every node periodically exchanges beacon messages to maintain the location information of its neighbors. A packet holder chooses the closest neighbor to be the next relay node. A right-hand rule is used for bypassing the void region after a void region is reached.

In dynamic network environments due to node mobility, node sleeping and link failures, to reduce packet losses, a forwarding node will sets up multi-backup next-hop nodes so that it can have alternative (backup) nodes to choose. For instance, the medium access control (MAC) layer will fail to deliver a packet when a primary next-hop node dies and becomes unreachable. After several retransmission failures, the MAC layer will drop the packet and notify the network layer. When receiving the notification, the routing protocol immediately selects a backup next hop and transmits the same packet, which is stored in the cache, down to the MAC layer. If the backup next hop also dies, such a retransmission mechanism will be repeated. When the node failure rate is higher than the threshold, such a multiple backup-node strategy along with data caching will severely increase network ETE delay, reduce bandwidth utilization and waste nodes energy due to unnecessary retransmissions.

On the other hand, the stateful routing intrinsically uses a ‘transmitter-oriented’ [13] approach based on the following steps: firstly, the next hop node is selected based on the neighbor information table, and then, the packet is transmitted forward to the selected node until the network reaches a predetermined number of transmission failures. Therefore, this routing suffers from several drawbacks in a highly dynamic network: (1) maintenance of neighbor information causes too much communication resource waste and results in significant energy consumption [20]; (2) neighbor information collection is often quickly outdated, which leads to frequent packet drops; and (3) the maintenance of neighbor information consumes scarce memory resource in WSNs.

### 2.2. Beaconless Geographic Routing

With the aim to overcome the weakness of conventional geographic routing schemes in dynamic network topology scenarios, the stateless routing protocols [6,15,16,18,21] have been proposed, such as contention-based [15], beaconless routing [16], etc. The beaconless routing schemes (BLR) are fully reactive, wherein every node transmits packets without the aid of beacons and neighbor information maintenance. A node will broadcast the packet it wants to transmit to its neighbors. The most suitable neighbor to be a relay node and further forward the packet is determined by a contention mechanism. The contention mechanism regulation of every neighbor determines a proper delay for further forwarding the packet based on the information of how well it is suited as the next-hop relay. The forwarding decision is determined based on the actual topology when forwarding packets. Therefore, beaconless routing schemes are robust to topology changes. However, the acknowledgment collision, which is one of the core issues that must be addressed, has a significant impact on the robustness of volunteer forwarding, latency and energy efficiency [22]. In order to avoid the acknowledgment collision among multiple potential forwarder candidates, contention timers are set to decide when to answer the packet holder. However, it is still not shown how to make PFs autonomously determine their acknowledgment precedence, while excessive message collisions and hop delay are controlled. To solve this problem, some protocols limit the selection of PFs within a so-called forwarding area with some typical shape, such as sector, Reuleaux triangle and circle. There are two major principles to partition forwarding areas: (1) every pair of PFs can hear each other’s replies in the same area; (2) the discrete dynamic forwarding delay (the waiting time before answering the packet holder) can be decreased while avoiding collisions.

### 2.3. Cooperative Communications

In reliable and energy-efficient routing (REER) [17], due to the cooperative multi-hop mesh structure [23,24], the relaying candidates are assigned during the multi-hop mesh route discovery and establishment procedure. In terms of algorithm design complexity and network operations [25,26], the pre-assigned scheme is chosen to be the simplest approach for relaying candidate selection. However, the pre-assigned relaying candidate selection scheme cannot deal with dynamic wireless channel variation. Furthermore, the relaying candidate selection is constructed fully independently of the data flow and incurs significant communication overhead when network status becomes better. By comparison, the RCR-selection is more adaptive in dynamic network environments [9,27].

## 3. Network Model

In the proposed network model, all sensor nodes are location-aware and equipped with the same radio transceiver. Their transmission range *R* is in the range $\left(0,R_{max}\right]$, where $R_{max}$ is the maximal transmission range. Every node is aware and knows the location of the sink, as well as its own location. Generally, the topology of sensor networks dynamically changes during the network operation, because of: (1) energy saving in the entire operation (sensor nodes may periodically switch to sleep mode) (in WSNs, there are generally two kinds of networks: always-on WSNs, wherein sensors are always awake, and duty-cycled WSNs, wherein sensors dynamically sleep and wake); and (2) possible unreliable links and node failures at any moment.

We model a multihop WSN with area size *S* by a graph G=(V,E) and a directed link (u,v)∈E if |uv|≤R. Therein, the set of vertices $V=\{v1,v2,\cdots ,v_{N}\}$ represents a set of N=|V| sensor nodes in the established network, and |uv| is the Euclidean distance between nodes *u* and *v*, which can communicate with each other directly without relaying.

### 3.1. Cooperative Nodes Search Region

**Figure 1:**
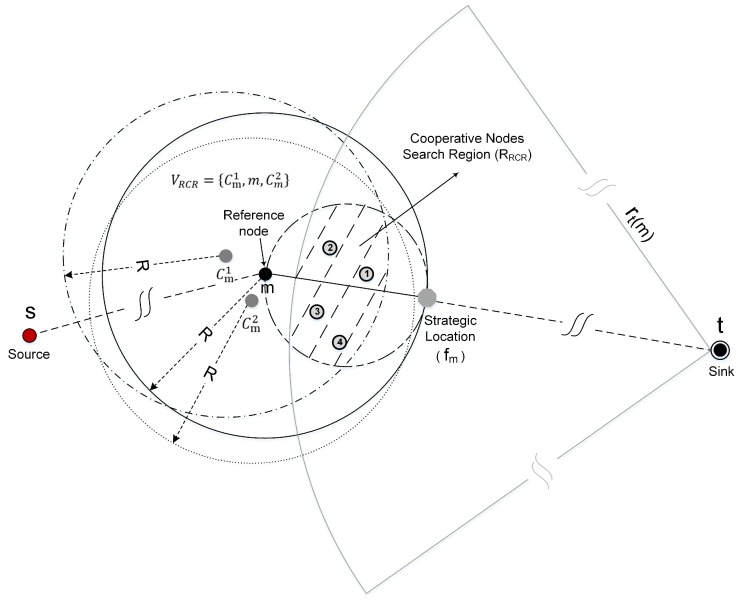
Illustration of the cooperative nodes search region.

**Definition** **1** (Cooperative nodes search region)**.** For a certain reference node m, $C_{m}=\left\{c_{m}^{1},c_{m}^{2},\cdots ,c_{m}^{k-1}\right\}$ represents its associated k−1 cooperative nodes. The search region to find the next-hop cooperative nodes is represented by $R_{RCR}$, which is defined as the overlapping area of the k circle regions with the centers of m, $c_{m}^{1}$, $c_{m}^{2}$, ⋯, $c_{m}^{k-1}$, whose radius is R. The radius of the circle region with the center of sink t is represented as $r_{t}\left(m\right)$, where $\left|mt\right|-R<r_{t}\left(m\right)<\left|mt\right|$, while the radius of the circle region with the center of the middle node between m and $f_{m}$ is $\frac{R}{2}$, where $f_{m}$ represents the strategic location as shown in Figure 1.

**Figure 2:**
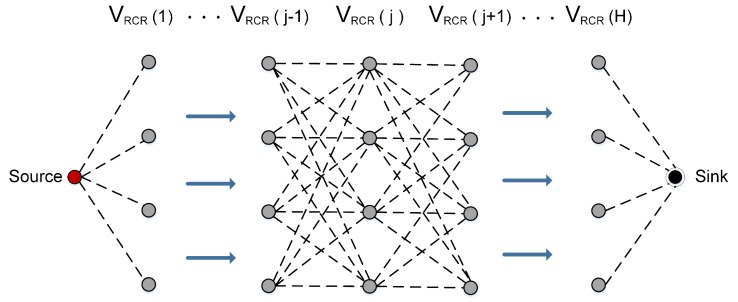
Cooperation between adjacent groups of cooperative nodes.

We use $V_{RCR}$ to represent the set of cooperative nodes in RCR as shown in Figure 2. Thus, $V_{RCR}$ is equivalent to the combination of *m* and $C_{m}$, i.e., $V_{RCR}=\left\{m,c_{m}^{1},c_{m}^{2},\cdots ,c_{m}^{k-1}\right\}$. In order to keep the consistency of the algorithm, we also represent the reference node *m* as $c_{m}^{0}$.

### 3.2. Selection of Cooperative Nodes

Within the search region of cooperative nodes, *k* nodes are selected as the cooperative nodes for the next hop. In our study, the ideal location for next-hop reference node *m* to select cooperative nodes for the next-hop is defined as follows:

**Definition** **2** (Strategic location for RCR-selection)**.** For a certain reference node m, the strategic location for selecting its next-hop cooperative nodes is represented by $f_{m}$, which is defined as a location on the straight line from m to the sink t, where the distance between m and $f_{m}$ is equal to R, i.e., $|mf_{m}|=R$.

By pure use of the location as a benchmark, the nodes located in the selection region whose distance to the strategic location is among the first *k* shortest value (the premise is to satisfy $|mf_{m}|=R$) will be selected as cooperative nodes of the next hop.

In order to achieve load balancing of the whole network [28,29], the residual energy of sensor nodes should be considered. We assume that every node starts with the same initial energy corresponding to the full battery capacity. The remaining battery capacities represented as current residual energy of the sensor nodes are discretized into integer-valued quantized-energy-levels ($L_{\mathrm{QE}}$). The criterion based on joint distance and residual energy is defined as follows:

**Definition** **3.** For a certain node u in the search region of cooperative nodes, $L_{QE}\left(u\right)$ represents its quantized-energy-level (residual energy) and $|uf_{m}|$ represents the distance between u and strategic location. The priority to be a cooperative node for the next hop of u is denoted by $Q_{u}$, which is defined by $Q_{u}=\sqrt{{\left(1-\frac{|uf_{m}|}{R}\right)}^{2}+{\left(\frac{L_{QE}\left(u\right)}{L_{max}}\right)}^{2}}$, where $L_{max}$ is the full battery level (the initial energy).

The closer the distance from *u* to $f_{m}$ is, the larger the value of $Q_{i}$ will be, whereas the higher $L_{\mathrm{QE}}\left(u\right)$ (the residual energy) of *u* is, the larger the value of $Q_{i}$ will be. Therefore, the nodes within the first *k* largest $Q_{i}$ will be selected as the cooperative nodes under this criterion.

### 3.3. Selection of the Reference Node for the Next Hop

Among the RCR, the cooperative node with the shortest distance to the strategic location will be selected as the reference node of the next hop.

### 3.4. RCR Update

The network operation time is divided into epochs, and each epoch is represented as *T*. In each epoch, the source node restarts RCR-selection by broadcasting a probe message. Then, the cooperative nodes list is updated based on the distance and residual energy of neighborhood nodes.

### 3.5. Multihop Cooperative Structure

Let $V_{RCR}\left(j\right)$ denote the set of $k=|V_{RCR}\left(j\right)|$ cooperative nodes (i.e., RCR) at the *j*-th hop; let  $V_{RCR}\left(j+1\right)$ denote the adjacent cooperative group of $V_{RCR}\left(j\right)$ a hop closer to the sink. Let $m_{j}$ and $m_{j+1}$ denote the reference nodes of $V_{RCR}\left(j\right)$ and $V_{RCR}\left(j+1\right)$, respectively. According to Definition 1, for given $u\in V_{RCR}\left(j\right)$, and $v\in V_{RCR}\left(j+1\right)$, |uv|≤R, and for $u_{1},u_{2}\in V_{RCR}\left(j\right)$ and $v_{1},v_{2}\in V_{RCR}\left(j+1\right)$, we get $|u_{1}v_{2}|\leq R$.

## 4. On-Demand ETE Reliability Model

### 4.1. Probability Model for Successful Delivery in One Hop

Assume the duty cycle is μ and the number of cooperative nodes is *K*, and the probability that *k* cooperative nodes are awake among RCR is represented by $P_{k}$. Then, $P_{k}$ can be calculated by Formula (1):

(1) $$\begin{array}{ccc} P_{k} & = & C_{K}^{k}\times \mu^{K-k}\times {\left(1-\mu \right)}^{k}. \end{array}$$

In that case, for *k* awake cooperative nodes, the probability of successful transmission at the current hop is represented by $W_{k}$, which can be calculated by Formula (2).

(2) $$\begin{array}{ccc} W_{k} & = & P_{k}\times \left(1-f^{k}\right)\\ & = & C_{K}^{k}\times \mu^{K-k}\times {\left(1-\mu \right)}^{k}\times \left(1-f^{k}\right). \end{array}$$

### 4.2. Probability Model for ETE Successful Data Delivery

If we use $P_{k}$ defined above for the *j*-th hop where *k* cooperative nodes are awake, the delivery failure ratio $f_{k}\left(j\right)$ is calculated by Formula (3).

(3) $$\begin{array}{ccc} f_{k}\left(j\right) & = & P_{k}\times f^{k}\\ & = & C_{K}^{k}\times \mu^{K-k}\times {\left(1-\mu \right)}^{k}\times f^{k}. \end{array}$$

Further, for *k* awake cooperative nodes, the probability of transmission failure at the current hop f(j) is defined by Formula (4).

(4) $$\begin{array}{ccc} f(j) & = & \sum_{k=1}^{k=K}f_{k}\left(j\right)\\ & = & f_{1}\left(j\right)+f_{2}\left(j\right)+\times f_{K}\left(j\right). \end{array}$$

Then, the successful delivery ratio at hop *j* is represented by W(j), and it is defined by Formula (5), where $C_{K}^{0}\mu^{K}$ represents that all nodes are sleeping when transmission has failed; $C_{K}^{1}\left(1-\mu \right)\times \mu^{K-1}\times f$ represents that only one node is awake; however, the node fails its transmission; $C_{K}^{2}{\left(1-\mu \right)}^{2}\times \mu^{K-2}\times f^{2}$ represents that two nodes are awake among RCR; however, both of the nodes fail in their transmissions, and so on. Similarly, $C_{K}^{K}{\left(1-\mu \right)}^{K}\times f^{K}$ represents that all cooperative nodes are awake, but all their transmissions have failed.

(5) $$\begin{array}{ccc} W(j) & = & 1-f(j)\\ & = & 1-f_{1}\left(j\right)-f_{2}\left(j\right)-\cdots -f_{K}\left(j\right)\\ & = & 1-C_{K}^{0}\mu^{K}-C_{K}^{1}\left(1-\mu \right)\times \mu^{K-1}\times f\\ & & -C_{K}^{2}{\left(1-\mu \right)}^{2}\times \mu^{K-2}\times f^{2}\\ & & -\cdots -C_{K}^{K-1}{\left(1-\mu \right)}^{K-1}\times \mu \times f^{K-1}\\ & & -C_{K}^{K}{\left(1-\mu \right)}^{K}\times f^{K}\\ & = & 1-{\left(\mu +\left(1-\mu \right)\times f\right)}^{k}. \end{array}$$

Then, the ETE reliability is defined by Formula (6).

(6) $$\begin{array}{ccc} W & = & W(1)\cdots W(j)\cdots W(H). \end{array}$$

For the sake of simplicity, we assume that the reliability is the same for all hops; thus:

(7) $$\begin{array}{ccc} W & = & W{\left(j\right)}^{H}. \end{array}$$

**Figure 3:**
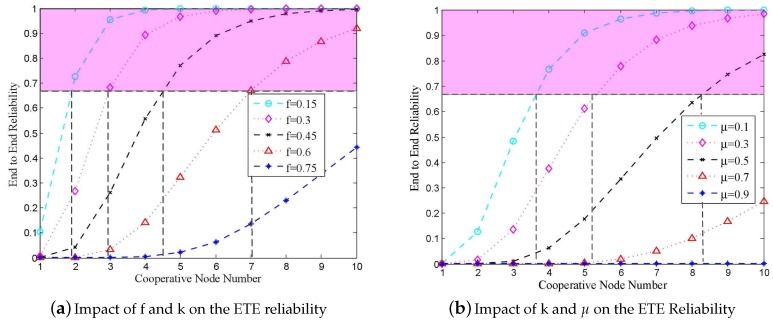
The impact of *f*, *k* and μ on reliability.

In order to verify the impact of *k* and *f* on *W* in Formula (7), we used MATLAB to perform the simulation, wherein we varied *k* from 1 to 10, while changing *f* from 0.15 to 0.75 with the step of 0.15, and μ was fixed to zero. In order to keep consistent with the simulation scenario, the hop count is set to 14. As shown in Figure 3a, the lower *f* is, the larger *k* and *W* are. In order to increase the reliability of packets, the FECcoding was used. After FEC package-level encoding, 67% successful FEC packet delivery can guarantee the recovery of original packets. Thus, based on the results presented in Figure 3a, the minimal *k* can be determined for certain *f*. According to the results presented in Figure 3a, where the shadow area represents the ETE reliability, which is larger than 67%, if *f* is equal to 0.3, *k* should be set to three, and if *f* is equal to 0.45, then the minimal *k* increases to five.

In Figure 3b, *k* changes from one to 10, μ changes from 0.1 to 0.9, *f* is set to 0.3, while the hop count is equal to 14. The curves show the impact of *k* and μ on *W*. Accordingly, when μ is smaller, *k* and *W* are larger. If FEC coding [29] is applied, then when μ = 0.1, *k* should be set to four, and when μ = 0.5, then *k* should be set to nine.

**Figure 4:**
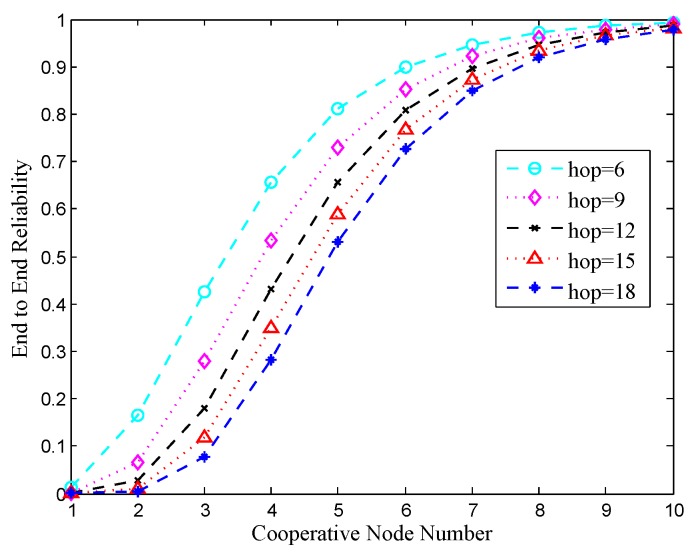
Impact of k and hop on ETE reliability.

In the simulations, we varied *k* from one to 10, and the hop count was changed from six to 18 with a step of three. The impact of *K* and hop count on *W* is shown in Figure 4. Typically, the larger *k* is, the smaller the hop count is and the higher the reliability is.

## 5. RCR Algorithm Design

**Figure 5:**
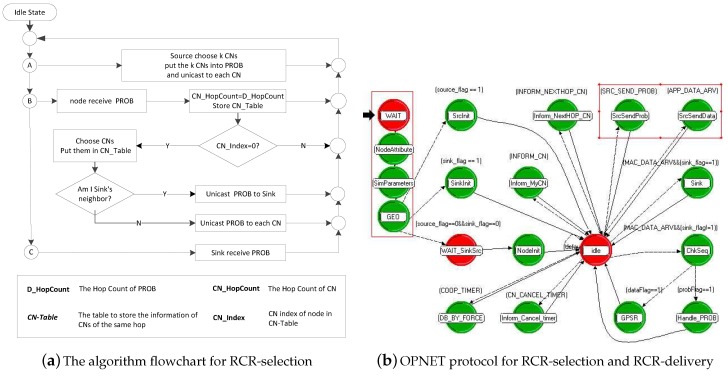
The algorithm flowchart and OPNET protocol for the RCR scheme.

The algorithm flowchart of RCR-selection is shown in Figure 5a, and the OPNET protocol for RCR-selection and RCR-delivery is shown in Figure 5b. Specifically, the pseudocode of the RCR scheme is presented in Algorithm 1.

**Figure 6:**
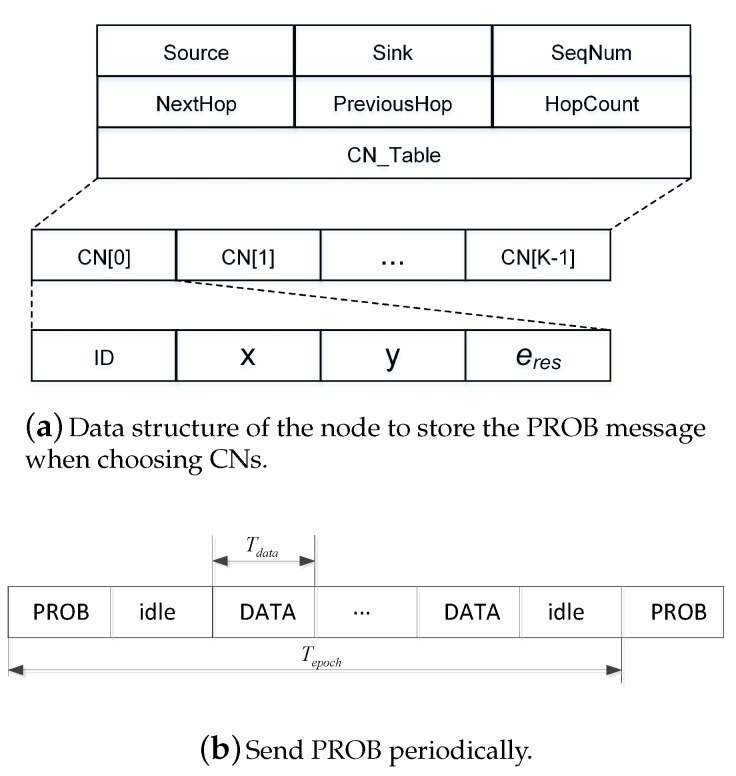
Probe (PROB) packet format and sending mechanism.

**Algorithm 1**
$C_{m}$ choosing algorithm.
**begin**
**notation**
  $n_{m}$ denotes the total number of neighbor nodes of m;   $V_{m}$ denotes the array of neighbor nodes of m;   *t* is the sink node;   $R_{RCR}$ denotes the cooperative nodes search region of m;   *Q* is array of priority for every neighbor nodes of m;   $C_{m}$ is array of cooperative nodes of m; **initialization**
**for** *i* = 0 to K−1 **do**   $Q_{Max}$ ← 0;    idx ← −1;    **for** *j* = 1 to $n_{m}$ **do**    **if** $V_{m}\left[j\right]$ is *t* **then**      break;     **end if**    **if** $(V_{m}\left[j\right]$ in $C_{m})$ OR $(V_{m}\left[j\right]$ not in $R_{RCR})$ **then**      continue;     **end if**    **if** $Q\left[j\right]>Q_{Max}$ **then**      $Q_{Max}$ ← Q[j];       idx ← *j*;     **end if**   **end for**   $C_{m}^{i}$ ← $V_{m}\left[idx\right]$;    **if** $C_{m}^{i}$ is *t* **then**    break;    **end if**  **end for**   Return $C_{m}$;


Figure 6a shows the data structure of the node to store the PROB message when choose CNs. The field named “CN_Table” is an array where the PROB information of which node received is stored, and each CN includes node ID, coordinates (x,y) and node residual energy e_res.

Figure 6b shows the time period of sending PROB and DATA. In order to prevent a few CNs from excessively taking the role of data forwarding, the CN list needs to be re-selected periodically to achieve load balancing. The re-selection of the CN list is activated by a source node to initiate a new PROB.

At each hop, the master CN will choose *k* CNs for its next hop and put them into the PROB message and then broadcasts the message. When a node receives the PROB message, it will judge whether it is in CN_Table or not. If the node is selected as a CN, its CN_Flag will be marked as one, and CN_HopCount is recorded. Especially, if its CN_Index is equal to zero, the node works as the role of master CN. This procedure repeats at every hop until the sink node receives the PROB message.

## 6. Performance Evaluation

We implemented the proposed RCR in three schemes, namely BLR, GPSR and REER, using the OPNET modeler and performed extensive simulations [30]. We built a wireless sensor network with a 1000 m × 500 m field where sensors were randomly deployed. In order to verify the scaling property of RCR, we set up 800 sensor nodes in this large-scale network. By default, we set *f* to 0.3, μ to 1, *k* to 5, *R* to 75 m and τ to 0.025 s. We used the same energy consumption model as in [31].

### 6.1. The Impact of f and K

**Figure 7:**
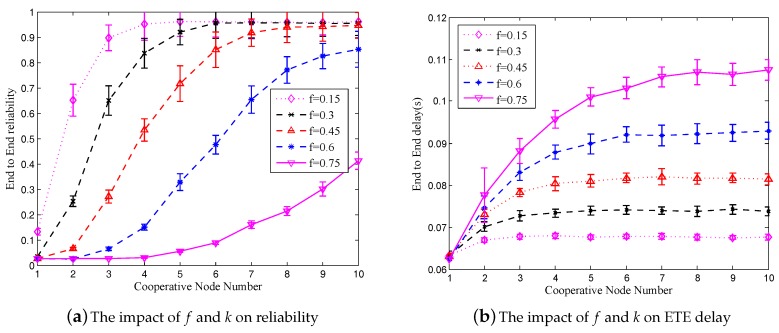
The impact of *f* and *k* on reliability and ETE delay in RCR.

In the simulations, the hop counts were set to 14 and μ was equal to zero. *k* is varied from one to 10, and *f* was varied from 0.15 to 0.75. Figure 7a shows the obtained results for the impact of *K* and *f* on reliability. The OPNET simulation results match with the theoretical results obtained by MATLAB, which are shown in Figure 3a.

According to the results presented in Figure 7b, wherein the impact of *k* and *f* on ETE delay is shown, the larger *f* is, the longer the ETE delay is. However, when the value of *f* is determined, the ETE delay converges to the stable value with the increase of *k*. Thus, we need to make a compromise between *k* and *f*.

### 6.2. The Impact of k and μ on Reliability and ETE Delay in RCR

**Figure 8:**
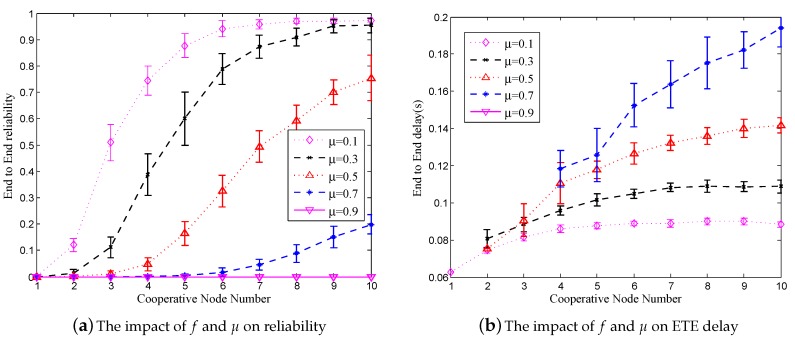
The impact of *f* and μ on reliability and ETE delay in RCR.

In the simulation whose results are presented in this section, *f* was fixed to 0.3 and *k* varied from one to 10. Figure 8a shows the impact of *f* and μ on reliability under the variations of μ from 0.1 to 0.9 with a step of 0.2. The simulation results are compatible with the theoretical results shown in Figure 3b. Moreover, the simulation results presented in Figure 8b show that the larger μ is, the longer the ETE delay is. Once μ is fixed, with the increase of *k*, the ETE delay converges to a certain value.

### 6.3. Performance Comparison among RCR, REER, BLR and GPSR

**Figure 9:**
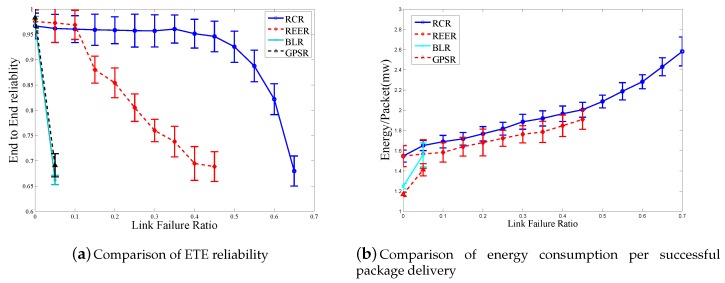
Comparison of ETE reliability and energy consumption with FEC.

On top of the data delivery schemes, we transferred a video sequence from the source node to the sink. Since the compressed video bit stream is sensitive to transmission errors because of the frame dependency, error control techniques such as forward error correction (FEC) were necessary to obtain the high reliability required by video services. We implemented the FEC coding scheme proposed in [29], and n−m redundant packets were generated to protect *m* data packets of a video frame. The size of each FEC packet was equal to the maximal size of data packets. If any *m* of *n* packets in the coding block were received by the sink, the corresponding video frame was successfully restored. In our simulation, three (n−m=3) FEC packets were used to protect six original video packets (m=3). We compared RCR with the other three algorithms in terms of reliability and energy consumption per pack, as shown in Figure 9. In the simulation, we changed *f* from zero to 0.95 with the step of 0.05. We set *k* in RCR to nine and r_value of REER to 65 m (i.e., the reference node at every hop had nine cooperative nodes on average).

In Figure 9a, the performances of four schemes, i.e., REER, BLR, GPSR and RCR, are compared in terms of reliability. With the increase of *f*, the reliability of GPSR decreases with the fastest speed among all schemes due to the lack of a mechanism for tackling packet loss. The BLR periodically re-selects the relay node using two forwarding mechanisms, namely the relay node-based forwarding and normal forwarding. During the selection of the relay node, broadcasting was utilized to achieve high reliability. However, once the relay node was selected, in the stage of normal forwarding, the same relay node was used for a certain period without the ability to antagonize fluctuations in channel quality. Thus, with the increase of *f*, the reliability of BLR decreases quickly. As indicated in BLR, a timer was set, and the time value was inversely proportional to the distance progress. However, in the simulation, the data collision among multiple nodes was hard to avoid because of the timer parameter, i.e., 0.04 s [16]. Thus, we implemented three hand-shakes including DATA, REPand SELin the protocol, which was adopted by REER [17].

Compared to the BLR, both REER and RCR select multiple relay nodes for every hop to achieve high reliability. In order to compare the RCR with the REER, we considered a scenario where *k* is set to nine. In the RCR, nine CNs were selected for each hop. However, the number of CNs is unstable in REER because r_value was first calculated according to *k*. Due to the uneven node distribution, the practical number of CNs based on a fixed r_value can be much smaller than nine. We varied r_value from 40 m to 75 m with a step of 5 m and found that the average number of CNs was nine when r_value was 65 m and the average hop count was 10.7. In comparison, the selection of the forwarding area in RCR was stricter than REER. For the same *k* (k=9), the average hop count reached 14.4 in RCR. Though RCR requires a higher hop count, the number of CNs was stable at each hop. Thus, the reliability was high and predictable. In the case when the average *k* was nine, the REER would have poor performance with lower reliability; because the bottleneck hop with an unpredictable small number of CNs reduced the whole ETE reliability.

**Figure 10:**
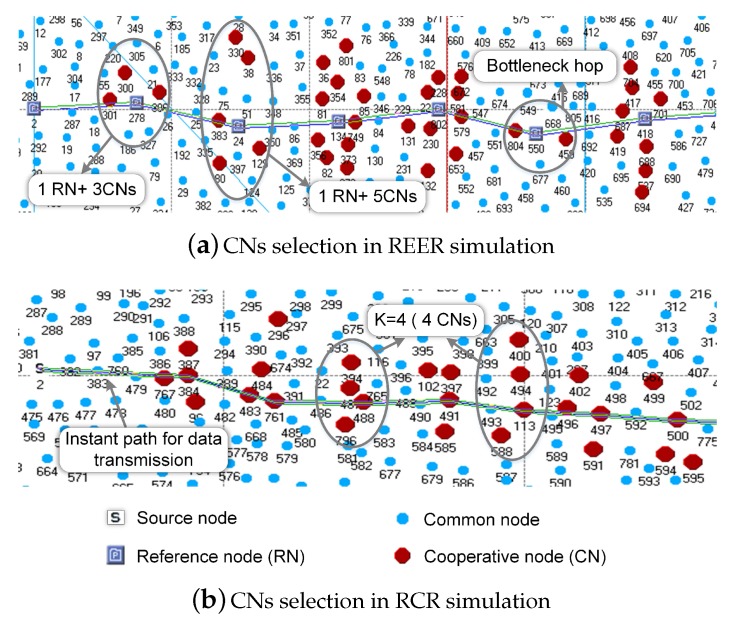
CNs select in simulation.

Figure 10 shows the snapshots of OPNET simulations for the same network scenario for the REER and RCR protocols. The number of cooperative nodes (packet forwarders) in hops in REER was unstable, as shown in Figure 10a. For instance, some hops had six cooperative packet forwarders, which included one reference node and five cooperative nodes, while the bottleneck hop only had three cooperative packet forwards, which was because the uneven node distribution in the fixed CN selection region for a given predetermined r_value causes an unbalanced number of CNs at every hop. In contrast, with the same average number of CNs, RCR produced the same number of CNs at each hop with the dynamic node distribution. Since the hop distance in RCR is adaptive to the QoS requirement for reliability, there were four CNs at each hop, as shown in Figure 10b.

Furthermore, we employed the automatic repeat request (ARQ) mechanism in RCR, while REER did not have that mechanism. With the increase of *f*, RCR exhibited higher reliability than REER.

Figure 9b shows the energy consumption of four schemes, wherein it can be seen that when *f* is larger than 0.6, the reliability of GPSR and BLR decreases to zero, so we can skip the statistics of energy consumption. The BLR can partially antagonize link failure, and the sink node receives more data packets with less energy consumption per successful packet delivery. In RCR and REER, the non-cooperative nodes enter sleep status during the data dissemination stage, thus exhibiting a low energy usage. For the same average *k*, the hop count of RCR is larger than that of REER and RCR has retransmitted. However, the use of ARQ in RCR incurs a higher energy consumption than that in REER.

Thus, RCR has a little bit higher energy consumption per successful packet delivery than REER, but it still has a good control for energy consumption.

In summary, RCR yields the highest reliability with considerable energy saving in most scenarios with various node distributions. Furthermore, *k* can be set to meet a certain specific requirement for reliability, while the adjustment of μ can guarantee energy consumption while satisfying QoS demands.

## 7. Conclusions

In this paper, we proposed a novel RCR scheme to exploit an on-demand number of cooperative nodes to facilitate the prediction of personalized end-to-end (ETE) reliability. In the proposed RCR scheme, we consider energy-efficient cooperative node sleeping and clustering problems in cooperative sensor networks where clusters of relay nodes jointly transmit sensory data to the next hop. Specific to the cooperative node clustering problem, we propose an optimal duty cycle for a given number of cooperative nodes when the network state is dynamic, which enables part of the cooperative nodes to switch into idle status for further energy saving. Simulation results show that the proposed RCR scheme controls the energy consumption while guaranteeing ETE reliability. In future research, we will continue to improve the relay node selection and data transmission mode while considering a real environment and new wireless communication technologies. 

## Figures and Tables

**Table 1:** Notation. RCR, reliability-aware cooperative routing.

Symbol	Definition
*s*	source node,
*t*	sink node,
*H*	hop count between the source node and the sink node,
*f*	link failure rate,
δ	node density,
*R*	transmission range,
|uv|	the distance between nodes *u* and *v*,
*K*	the total number of cooperative nodes at each hop,
*k*	the number of awake cooperative nodes at each hop,
RCR	reliability-aware cooperative routing,
ρ	RCR construction cost per time,
$T_{epoch}$	RCR construction refreshing interval,
μ	duty cycle of sensors,
$P_{k}$	the probability with *k* number of
	awake cooperative nodes among RCR,
$m_{h}^{i}$	reference node at hop *i*, or $C_{m_{i}}^{0}$
$c_{m_{i}}$	$\left\{c_{m_{i}}^{1},c_{m_{i}}^{2},\ldots ,c_{m_{i}}^{k-1}\right\}$,
	the k−1 cooperative neighbor nodes of $c_{m_{i}}^{0}$,
$R_{RCR}$	cooperative nodes search region,
$R_{mt}$	the disk centered at sink *t* with radius $r_{t}\left(m\right)$
	where $\left|mt\right|-R<r_{t}\left(m\right)<\left|mt\right|$,
$R_{mf}$	the disk centered at the middle node
	between *m* and $f_{m}$ with radius $\frac{R}{2}$,
$n_{m}$	the total number of neighbor nodes of m,
$V_{RCR}\left(i\right)$	$\left\{m_{h}^{i},C_{m_{i}}^{1},C_{m_{i}}^{2},\ldots ,C_{m_{i}}^{k-1}\right\}$,
	the cooperative nodes at hop *i*,
$L_{QE}$	energy level of sensor node,
$W_{k}\left(i\right)$	given *k* available cooperative nodes,
	successful delivery ration at hop *i*,
*W*	end-to-end (ETE) reliability

**Table 2:** Comparison of greedy perimeter stateless routing (GPSR), beaconless routing schemes (BLR), reliable and energy-efficient routing (REER) and RCR.

Items	GPSR	BLR	REER	RCR
Categories	Stateful geo-routing protocol	Stateless routing protocol	Reliable and energy-efficient routing	Reliability-aware cooperative routing
Features	Waste communication resource; significant energy consumption	Acknowledgment collision; worse robustness of forwarding, latency and energy efficiency	Relaying candidate selection is independent from data flow; significant communication overhead	Adaptive in dynamic network environments; control energy consumption; guarantee ETE reliability
Related Works	Receiver-oriented load-balancing and reliable routing in wireless sensor networks [13]; GPSR: greedy perimeter stateless routing for wireless networks [14]	Contention-based forwarding for mobile ad hoc networks [15]; BLR: Beacon-Less Routing Algorithm for Mobile Ad Hoc Networks [16]	Reliable and energy-efficient routing protocol in dense wireless sensor networks [17]	QoS-aware distributed adaptive cooperative routing in wireless sensor networks [18]; Cooperative communication in wireless networks [19]
